# The Remineralization Potential of Silver Diamine Fluoride, ReMin Pro, Bioactive Glass, and Grape Seed Extract on Artificial Enamel Lesions: An In Vitro Study

**DOI:** 10.3390/biomimetics11100703

**Published:** 2026-10-03

**Authors:** Senem Gündüz, Ömer Çellik

**Affiliations:** Department of Restorative Dentistry, Faculty of Dentistry, Dicle University, 21280 Diyarbakır, Türkiye; dtomercelik@gmail.com

**Keywords:** remineralization, enamel, surface microhardness, bioactive glass, grape seed extract

## Abstract

**Objective**: Remineralization is an important strategy for preventing the progression of early enamel caries and restoring the mineral content of demineralized enamel. Various remineralizing agents, including fluoride-based materials, hydroxyapatite, bioactive glass, and natural compounds, have been investigated for their potential to promote enamel remineralization. However, evidence directly comparing the remineralization efficacy of these different agents under standardized experimental conditions remains limited. Therefore, a comparative evaluation of these materials may provide clinically relevant information for the management of early enamel lesions and contribute to the development of biomimetic strategies that aim to restore the structure and properties of damaged dental tissues. Accordingly, this study aimed to comparatively evaluate the remineralization potential of silver diamine fluoride, hydroxyapatite–xylitol, nano-sized bioactive glass, and grape seed extract on artificially induced enamel lesions. **Materials and Methods**: Fifty-five caries-free human premolars were used in this in vitro study. Fifty enamel specimens were randomly assigned to five groups (*n* = 10): 38% silver diamine fluoride (Riva Star), hydroxyapatite–xylitol (Remin Pro), nano-sized bioactive glass (Bone G Active), 10% grape seed extract (Immu-Nat), and deionized water (control). Five additional specimens were analyzed using scanning electron microscopy (SEM). All specimens underwent a 14-day pH cycling regimen. Remineralization was evaluated using Vickers surface microhardness measurements at baseline, after demineralization, and after remineralization. Surface morphology was assessed by SEM. Data were analyzed using a one-way ANOVA and Bonferroni post hoc tests (*p* < 0.05). **Results**: All experimental groups showed significantly higher microhardness values compared with the control group (*p* < 0.05). The greatest increases were observed in the Riva Star, Remin Pro, and bioactive glass groups. Although grape seed extract demonstrated a lower remineralization effect, it significantly improved microhardness compared with the control group (*p* < 0.05). SEM analysis revealed more pronounced surface morphological changes in the Riva Star and ReMin Pro groups. **Conclusions**: Silver diamine fluoride, Remin Pro, and bioactive glass demonstrated promising remineralization potential, whereas grape seed extract exhibited a supportive but limited effect.

## 1. Introduction

In dentistry, the primary objective is to preserve oral health, prevent the development of dental caries, and, in the presence of existing carious lesions, to prioritize non-invasive or minimally invasive treatment approaches [1]. Under acidic conditions, enamel mineral dissolves, resulting in changes in its microstructure and optical properties and leading to the development of initial carious lesions, which clinically appear as white opaque areas [2,3]. When the environmental pH increases, mineral ions can redeposit onto the enamel surface, a process known as remineralization. Demineralization and remineralization occur continuously in the oral environment, and when this dynamic balance shifts toward mineral loss, caries develop. With advances in technology, there has been a growing interest in the development of materials with various compositions that can reduce demineralization and enhance mineral gain in dental hard tissues [2,4].

Fluoride-containing preparations are among the most commonly used materials in the prevention of dental caries [5,6]. They exert this effect through various mechanisms that reduce demineralization and enhance remineralization [7,8]. However, the inability of fluoride ions to directly initiate the formation of well-organized apatite crystals limits their use for biomimetic remineralization [9]. Therefore, in line with advances in material science and nanotechnology, hybrid dental materials with diverse compositions have been developed. Bioactive materials such as nano-hydroxyapatite, self-assembling peptides, casein phosphopeptide–amorphous calcium phosphate (CPP–ACP), protein-based structures, giomer technologies, and smart delivery systems have gained prominence due to their active role in supporting the remineralization process [10,11]. These approaches are also closely related to biomimetic remineralization, which aims to reproduce or support the natural processes involved in the formation and regeneration of dental hard tissues. In particular, the use of hydroxyapatite and mineral ion-releasing materials may contribute to the formation of apatite-like mineral phases that resemble the mineral composition of natural enamel, providing a biomimetic basis for the development of strategies for enamel remineralization [12,13].

Silver diamine fluoride (SDF), bioactive glass, hydroxyapatite-based materials, and grape seed extract (GSE) have been investigated for enamel remineralization through different mechanisms. SDF may reduce mineral dissolution and stabilize demineralized enamel through its high fluoride content and antimicrobial activity [14,15]. Bioactive glass promotes mineral deposition through the release of calcium, phosphate, and silicate ions and the subsequent formation of an apatite-like layer on the enamel surface [16,17]. ReMin Pro, containing hydroxyapatite, fluoride, and xylitol, may support remineralization through direct mineral supplementation and fluoride-mediated mineral stabilization [18,19]. In contrast, GSE contains polyphenolic compounds that may interact with the organic matrix and facilitate mineral ion binding, providing a potentially different pathway for supporting mineralization [20,21].

The efficacy of remineralizing materials with different chemical compositions and mechanisms of action has been investigated using various in vitro models. Previous studies have reported differences in enamel remineralization among materials containing hydroxyapatite, bioactive glass, fluoride, and calcium phosphate [22,23]. More recent studies have likewise demonstrated variable remineralization performance among bioactive glass-, hydroxyapatite-, and fluoride-containing formulations [24,25]. Grape seed extract (GSE), which is rich in proanthocyanidins, has also been investigated as a natural remineralizing agent, with recent in vitro studies demonstrating beneficial effects on enamel microhardness and lesion characteristics [26,27]. However, the reported efficacy of GSE varies according to the experimental model and treatment conditions. In addition, a recent systematic review and meta-analysis reported substantial methodological heterogeneity among in vitro enamel remineralization studies with respect to lesion preparation, remineralization protocols, evaluation methods, and material characteristics [28]. These methodological differences may limit direct comparisons among remineralizing materials and highlight the importance of evaluating agents with distinct mechanisms of action under standardized experimental conditions.

In order to accurately evaluate remineralization, mineral changes occurring in artificial caries lesions should be assessed using multiple analytical methods [28]. For this purpose, various methods such as microhardness testing, scanning electron microscopy (SEM), energy-dispersive X-ray spectroscopy (EDS), and transverse microradiography (TMR) are used in the literature to evaluate surface and subsurface mineral gain. Combining both quantitative and qualitative analysis methods may provide a more comprehensive understanding of the remineralization process [29,30].

Although the remineralizing effects of silver diamine fluoride, hydroxyapatite-based formulations, bioactive glass, and grape seed extract have been investigated individually or in selected combinations, comparative evidence for these materials with distinct mechanisms of action under standardized experimental conditions remains limited. Therefore, this study aimed to compare the effects of 38% silver diamine fluoride, hydroxyapatite–xylitol, nano-bioactive glass, and grape seed extract on demineralized enamel using an artificial caries model and a 14-day pH cycling protocol. Enamel microhardness and surface morphology were assessed to determine the extent of enamel recovery following treatment. The findings provide comparative data on the effectiveness of these approaches with different biological and mineralization mechanisms and may help inform material selection for preventive dental applications.

The null hypothesis of this study was that there would be no statistically significant difference in the remineralization efficacy of different agents applied to enamel surfaces with artificially induced carious lesions.

## 2. Materials and Methods

### 2.1. Sample Size Calculation

Sample size was determined using G*Power software (version 3.1.9.7; Heinrich Heine University Düsseldorf, Germany). An a priori power analysis was performed using a one-way analysis of variance (ANOVA) model for five groups. Based on an effect size of f = 0.60, derived from a previous study [31]; a significance level of α = 0.05; and a statistical power of 85% (1 − β = 0.85), the minimum required sample size was calculated as 45 specimens (*n* = 9). To ensure the equal allocation of specimens among the five experimental groups, 50 specimens were included in the microhardness analysis (*n* = 10). In addition, one specimen from each group was reserved for scanning electron microscopy analysis, resulting in a total of 55 human premolar teeth.

### 2.2. Sample Preparation

**Figure 1:**
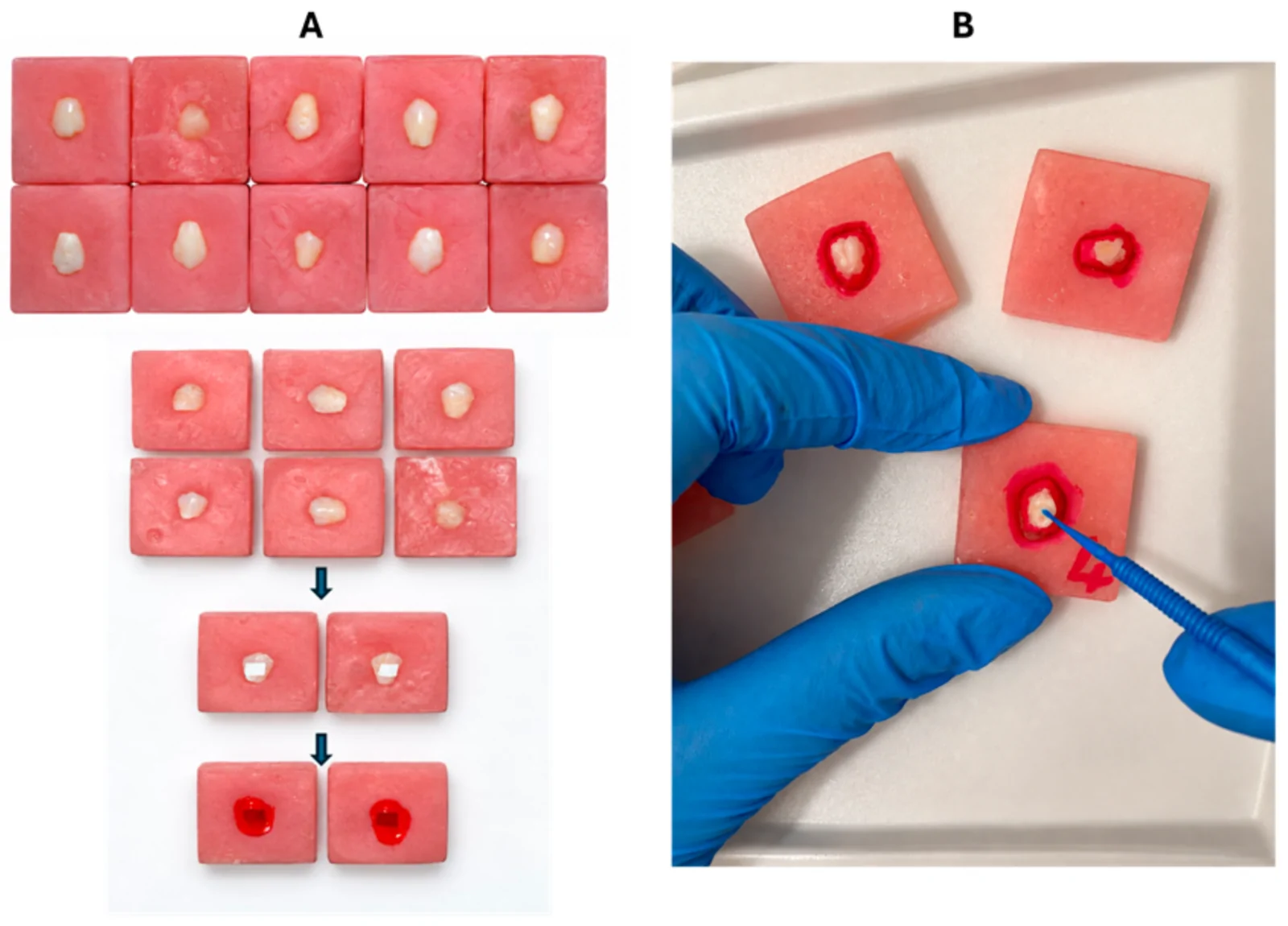
Experimental procedure. (**A**) Preparation and isolation of enamel specimens at different stages of experimental procedure. (**B**) Application of experimental agent to enamel surface using microbrush.

Enamel samples were obtained from 55 caries-free human premolars extracted for orthodontic or periodontal reasons (*n* = 11). After the removal of blood and tissue remnants, the teeth were stored in distilled water containing 0.1% thymol at +4 °C until the laboratory procedures. The middle third of the buccal enamel surface was selected for evaluation due to its relatively flat morphology. The crown and root portions were separated using a diamond disk under water cooling, and the crown segments were sectioned mesiodistally. Enamel samples were embedded in acrylic resin (Imicryl, Konya, Turkey) using silicone molds (2 × 2 × 2 cm), with the buccal surfaces exposed, and randomly assigned to five experimental groups. The enamel surfaces were polished with 200–1200-grit silicon carbide papers to obtain standardized surfaces. A 2 × 2 mm area was defined on each sample using adhesive tape, and the remaining surface was coated with two layers of acid-resistant varnish to prevent demineralization (Figure 1).

### 2.3. Demineralization Solution

The demineralization solution was prepared to create controlled mineral loss on the enamel surface. The solution consisted of the following components:

- A total of 2.0 mmol/L Ca(NO_3_)_2_·4H_2_O—calcium nitrate tetrahydrate.
- A total of 2.0 mmol/L NaH_2_PO_4_·2H_2_O—sodium dihydrogen phosphate dihydrate.
- A total of 75 mmol/L acetate buffer.

The pH of the prepared solution was measured and monitored, with adjustments made to 4.4 using 1.0 M KOH when necessary.

### 2.4. Remineralization Solution

The remineralization solution was prepared to promote mineral gain in demineralized enamel tissue. The composition of the solution was as follows:

- A total of 1.5 mmol/L Ca(NO_3_)_2_·4H_2_O—calcium nitrate tetrahydrate.
- A total of 0.9 mmol/L Na_2_HPO_4_·2H_2_O—sodium hydrogen phosphate dihydrate.
- A total of 150 mmol/L KCl—potassium chloride.
- A total of 20 mmol/L Tris buffer.

The pH value of the prepared solution was measured using a pH meter, and when necessary, it was adjusted to pH 7.0 by adding 1.0 M KOH.

The composition and concentrations of the demineralization and remineralization solutions were selected based on previously reported in vitro pH cycling models [32].

### 2.5. Artificial Carious Lesion Formation

To create artificial initial caries lesions, each sample was placed in a separate container with 10 mL of demineralizing solution and stored at 37 °C for 96 h [33]. Following the demineralization procedure, the samples were rinsed with distilled water and dried.

### 2.6. pH Cycling

**Figure 2:**
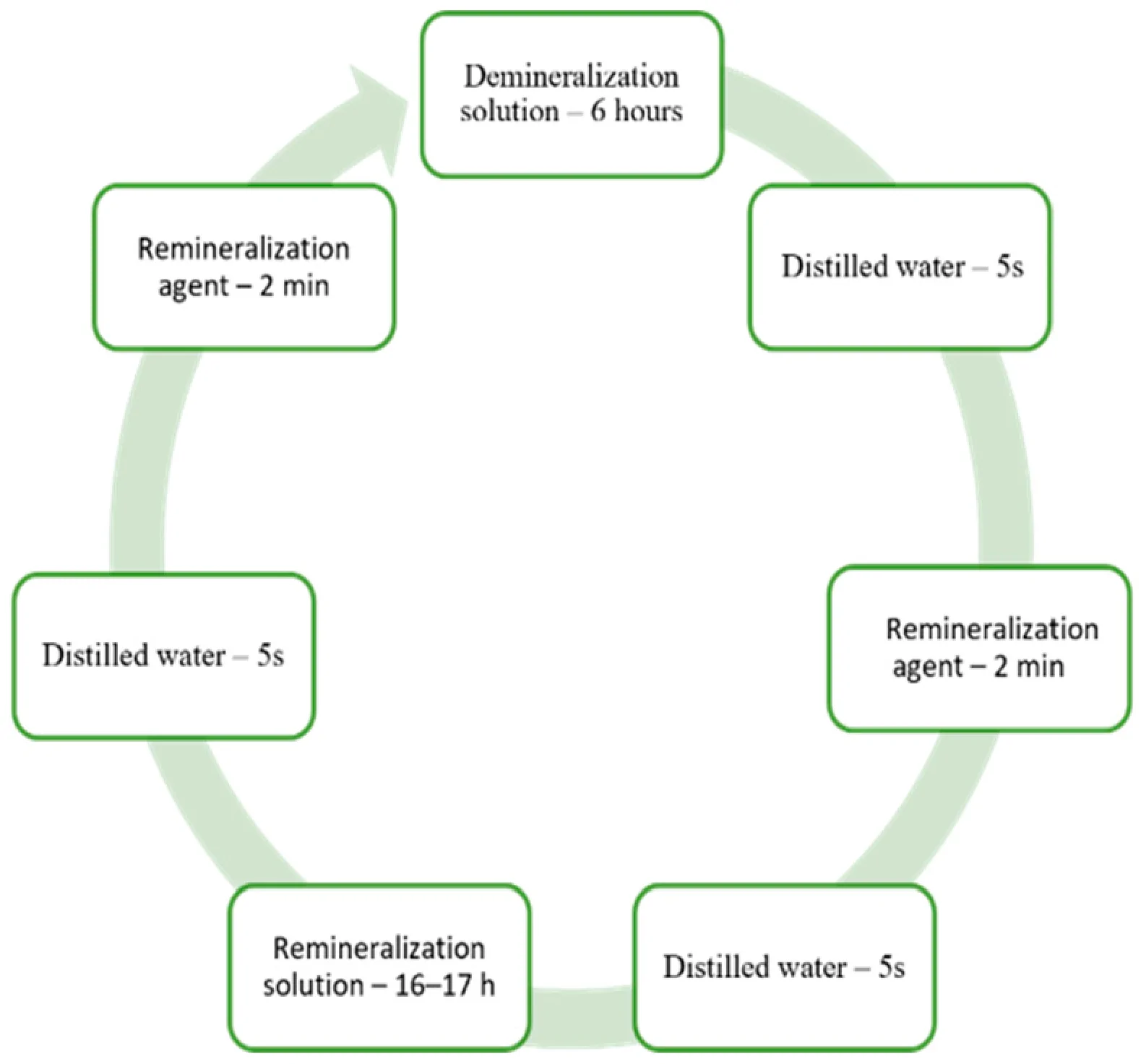
pH cycle.

To simulate pH changes in the oral environment, the samples were subjected to a daily pH cycle. In each 24 h cycle, the samples were kept in the remineralization solution (pH ≈ 7) for 18 h and in the demineralization solution (pH ≈ 4.3) for 6 h [29] (Figure 2).

The remineralizing agent was applied in two separate 2 min stages, following a previously reported in vitro remineralization protocol [34].

### 2.7. Formation of Experimental Groups

Group 1: Silver diamine fluoride (SDF).

Group 2: Xylitol + hydroxyapatite (REMIN-PRO).

Group 3: Nano-sized bioactive glass (prepared by sol–gel method).

Group 4: Grape seed extract (GSE).

Group 5: Control group with deionized water.

### 2.8. Remineralization Protocol

The specimens were treated twice daily with their respective remineralizing agents according to the manufacturers’ instructions, as described below. After each application, the specimens were rinsed with deionized water, and the solutions were renewed daily.

Silver diamine fluoride (Riva Star, SDI Limited, Victoria, Australia) was applied in two steps; the 38% solution was applied to the lesion area and left for 1 min, followed by the application of a second solution containing potassium iodide to complete the reaction. ReMin Pro (VOCO GmbH, Cuxhaven, Germany) was applied as a thin layer on the tooth surface and left for 2 min. Bioactive glass (Bone G Active, Meta Bioengineering, İstanbul, Türkiye) was mixed with distilled water to obtain a gel form and applied to the lesion surface, and excess material was removed after 2 min. Grape seed extract (10%; Immu-Nat, Muğla, Turkey) was applied twice daily with a microbrush during the pH cycling period, and each application lasted for 2 min. The samples were subjected to pH cycling between all applications. The composition and manufacturer information of the remineralization agents are presented in Table 1.

### 2.9. Microhardness Measurements

The surface microhardness of the specimens was measured using a Vickers microhardness tester (Shimadzu HMV-2, Kyoto, Japan). These measurements were performed at three time points: at baseline, after artificial carious lesion formation by demineralization, and after the completion of the 14-day pH cycling regimen following the application of the remineralization agents. Vickers microhardness values were calculated by measuring the diagonal lengths of the indentations produced under a 300 g load applied for 15 s. Three measurements were obtained from each specimen, and their mean value was used as the final Vickers microhardness value. To minimize edge effects, the peripheral areas of the specimens were excluded from the measurements.

### 2.10. SEM Analysis

Surface morphology was evaluated by scanning electron microscopy (SEM; FEI Quanta 250 FEG, FEI Company, Hillsboro, OR, USA). One representative specimen from each group was examined at three stages, baseline, after artificial carious lesion formation, and after the completion of the 14-day pH cycling regimen, and the surface morphological changes were recorded.

### 2.11. Statistical Analysis

The data were analyzed using SPSS (Statistical Package for the Social Sciences) for Windows, version 25.0 (IBM Corp., Armonk, NY, USA). Data normality was assessed using the Kolmogorov–Smirnov test. For normally distributed data, a one-way analysis of variance (ANOVA) was used to compare more than two independent groups, whereas a repeated-measures ANOVA was used for comparisons of more than two dependent measurements. When significant differences were detected, Bonferroni post hoc tests were performed for pairwise comparisons. Descriptive statistics were expressed as means ± standard deviations, and statistical significance was set at *p* < 0.05.

## 3. Results

Microhardness values were evaluated at baseline, after artificial caries lesion formation, and following remineralization. The corresponding measurements are presented in Table 2. A statistically significant difference was observed among the groups at baseline (*p* < 0.05), with the GSE group showing significantly lower microhardness than the control. Following artificial caries lesion formation, no significant differences were detected among the groups (*p* > 0.05). After remineralization, a significant difference was observed among them (*p* < 0.05); the control group showed significantly lower microhardness than all other groups, whereas no significant differences were found among SDF, ReMin Pro and bioactive glass.

Comparisons of the within-group changes from baseline to demineralization, demineralization to remineralization, and baseline to remineralization are presented in Table 3. Microhardness values decreased significantly after demineralization compared with the baseline in all groups (*p* < 0.001). Following remineralization, the SDF, ReMin Pro and bioactive glass groups showed no significant difference compared with baseline values, whereas the GSE group remained significantly different from the baseline (*p* < 0.05). In the control group, no significant difference was observed between demineralization and remineralization values, and both remained significantly lower than baseline values (*p* < 0.001).

**Figure 3:**
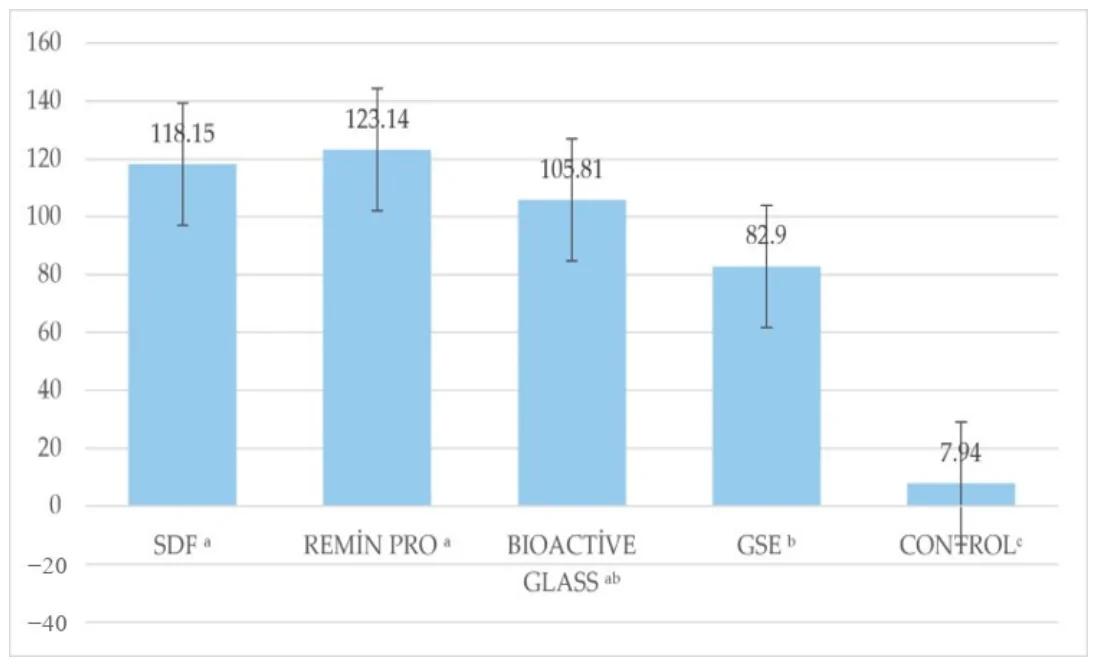
Changes in microhardness following remineralization after demineralization across the experimental groups. (Different superscript letters indicate significant differences (*p* < 0.05)).

A significant difference was observed among the groups in the increase in microhardness following remineralization after demineralization (*p* < 0.05), as shown in Figure 3. Bonferroni post hoc analysis showed that all experimental groups exhibited significantly greater increases in microhardness than the control group (*p* < 0.05). No significant differences were observed among bioactive glass, Remin Pro, and SDF (*p* > 0.05). The Remin Pro and SDF groups showed significantly greater increases than GSE (*p* < 0.05), whereas the difference between bioactive glass and GSE was not significant (*p* > 0.05).

### SEM Analysis Results

**Figure 4:**
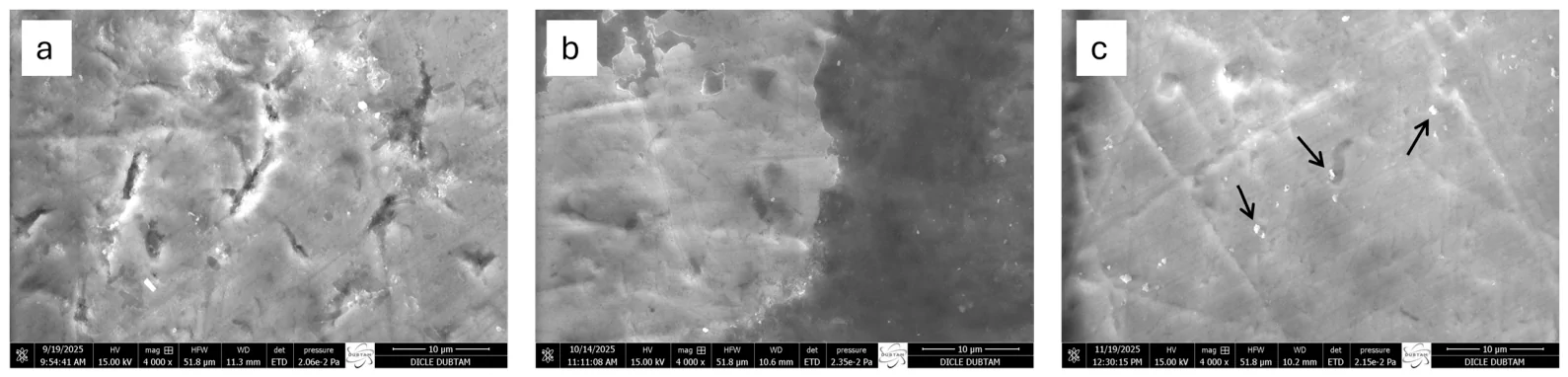
SEM images of the silver diamine fluoride (SDF) group at three stages: (**a**) baseline, (**b**) demineralization, and (**c**) remineralization. Arrows indicate areas with visible deposits on the enamel surface.

**Figure 5:**
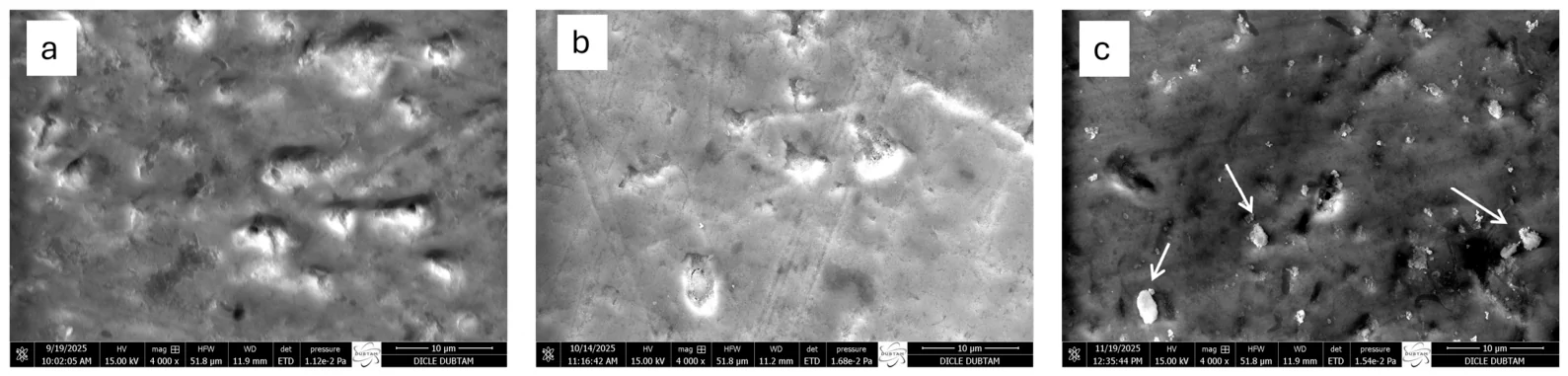
SEM images of the Remin Pro group at three stages: (**a**) baseline, (**b**) demineralization, and (**c**) remineralization. Arrows indicate areas with visible deposits on the enamel surface.

**Figure 6:**
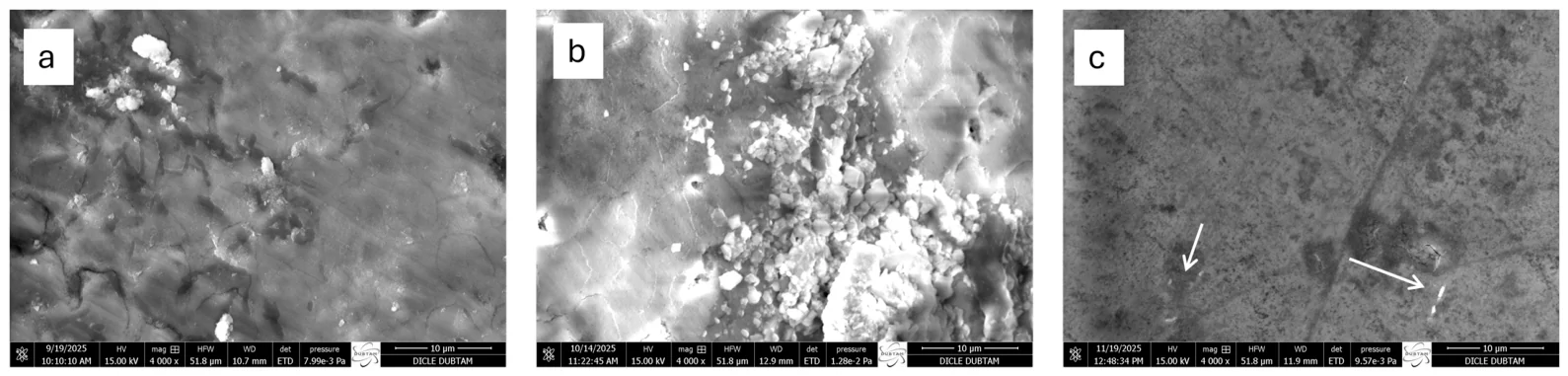
SEM images of the bioactive glass group at three stages: (**a**) baseline, (**b**) demineralization, and (**c**) remineralization. Arrows indicate areas with visible deposits on the enamel surface.

**Figure 7:**
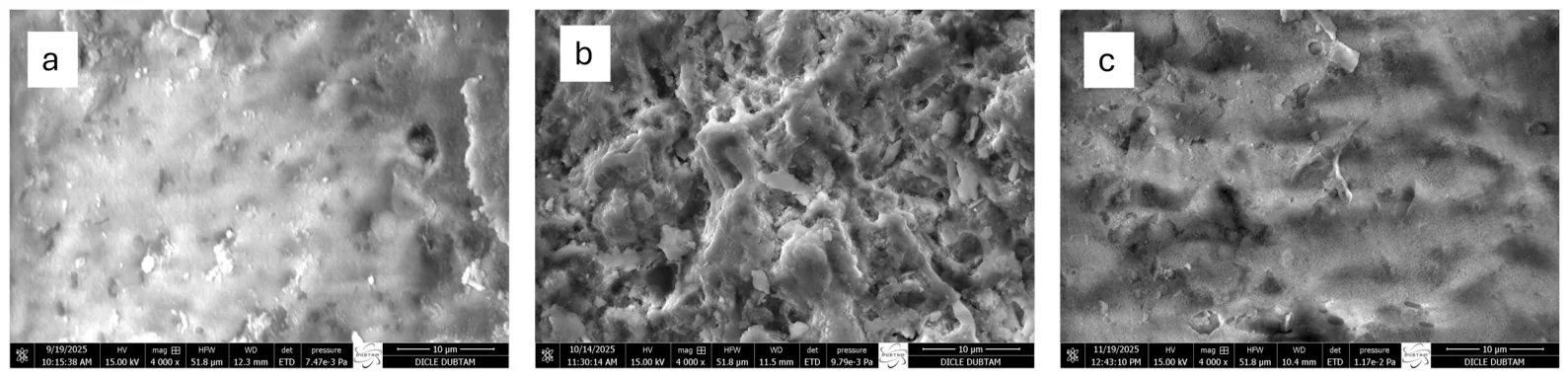
SEM images of the grape seed extract group at three stages: (**a**) baseline, (**b**) demineralization, and (**c**) remineralization.

**Figure 8:**
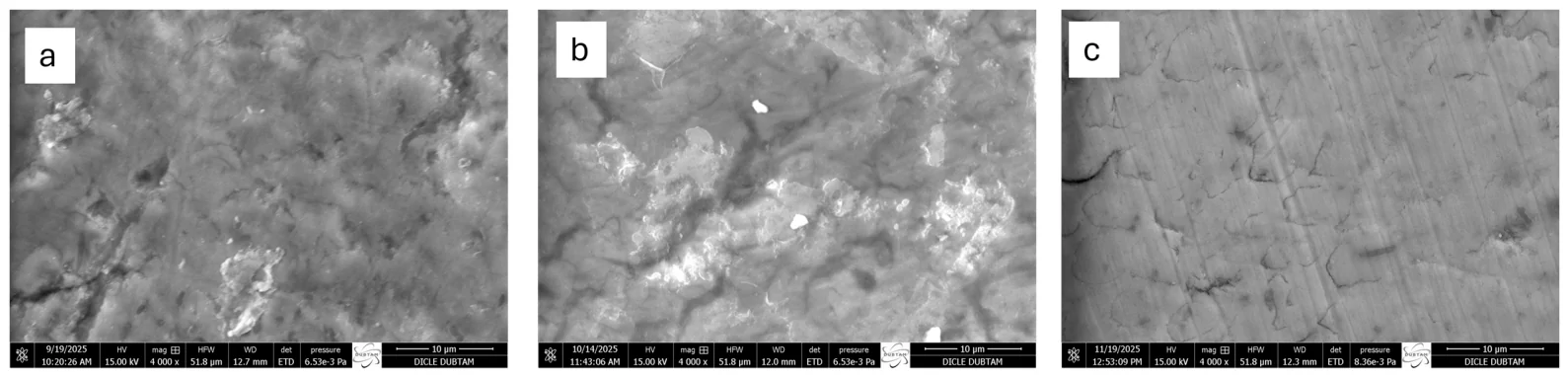
SEM images of the control group at three stages: (**a**) baseline, (**b**) demineralization, and (**c**) remineralization.

The SEM images obtained at baseline, after demineralization, and following remineralization are presented in Figure 4, Figure 5, Figure 6, Figure 7 and Figure 8. At baseline, the enamel surfaces exhibited a relatively smooth, homogeneous, and compact morphology. After demineralization, pronounced morphological changes were observed, with increased surface irregularity and heterogeneity, accompanied by visible microporosities and surface depressions. Following remineralization, varying degrees of surface morphological improvement were observed among the experimental groups. In the SDF and Remin-Pro groups, granular mineral deposits were observed on the enamel surface, which may be associated with calcium–phosphate-rich mineral precipitation. However, these deposits were localized and did not form a homogeneous surface layer, and surface regularization remained limited. In the bioactive glass and GSE groups, the surface morphology appeared more regular and compact, with a reduction in visible microporosity compared with the demineralized condition. In contrast, the control group retained the irregular and rough surface morphology observed after demineralization, with evident microporosities and surface depressions and no apparent mineral deposition or surface coverage.

## 4. Discussion

In this study, the remineralization capacities of silver diamine fluoride, ReMin Pro, bioactive glass, and grape seed extract were compared using artificially induced initial enamel caries lesions. Among the experimental groups, the most pronounced increase was observed in the silver diamine fluoride (SDF), ReMin Pro, and bioactive glass groups, indicating that these materials had greater remineralization potential than the grape seed extract and control groups. Although the increase in microhardness in the grape seed extract group was more limited than in the other experimental groups, it was higher than that observed in the control group. Accordingly, the null hypothesis was rejected because significant differences in microhardness recovery were observed among the groups.

An in vitro pH cycling model was used to evaluate the effects of different remineralization agents on initial enamel lesions under standardized experimental conditions [35]. This model simulates the daily pH fluctuations occurring in the oral environment, allowing the demineralization and remineralization processes in enamel to be investigated under controlled conditions and different remineralization agents to be evaluated comparatively [4]. Previous studies have reported that lesions produced under appropriate pH conditions exhibit superficial mineral loss similar to that observed in natural initial caries lesions, supporting the use of this model for the comparative evaluation of different remineralization agents [36,37]. In the present study, although baseline microhardness values differed among the groups, no significant differences were observed following demineralization. This finding indicates that the demineralization procedure resulted in a relatively standardized lesion condition for the subsequent comparison of the remineralization effects of the tested materials.

Silver diamine fluoride (SDF) is considered an effective agent for the management of early caries lesions due to its high fluoride content and the antibacterial activity of silver ions [38]. Its remineralizing effect is associated with the ability of fluoride ions to reduce hydroxyapatite dissolution during demineralization and promote the formation of more acid-resistant fluorapatite-like structures [8]. The sustained availability of low levels of fluoride facilitates the redeposition of calcium and phosphate ions onto demineralized enamel surfaces, resulting in increased surface hardness [39]. Consistent with these mechanisms, previous in vitro studies have reported substantial remineralization effects following SDF application [40], and high-concentration SDF applications have been shown to increase mineral density and remineralization depth [41]. The effectiveness of SDF is not limited to mineral gain; it is also known that silver ions can slow the progression of caries by inhibiting bacterial metabolism. This dual mechanism provides a significant advantage, particularly in supporting the remineralization process in early-stage lesions [42]. The effectiveness of SDF in caries control has also been demonstrated in clinical studies [14,43]. It has been reported that 38% SDF applied at regular intervals is effective in reducing caries progression and preventing the development of new caries in children [14]. The significant increase in microhardness observed in the SDF group in the present study is consistent with these previous findings. Nevertheless, the clinical application of SDF should also be considered in relation to its esthetic limitations. Silver-related discoloration is a well-recognized consequence of SDF application and may influence its clinical acceptance, particularly in esthetically important areas [44]. Therefore, future studies evaluating SDF-based remineralization should consider color change together with mineral recovery to provide a more comprehensive assessment of its clinical effects.

The significant increase in microhardness observed in the ReMin Pro group compared with the control group demonstrates the remineralization potential of this material in artificially induced initial enamel caries lesions. This effect is associated with the composition of ReMin Pro, which contains hydroxyapatite, fluoride, and xylitol [18,45]. Hydroxyapatite particles provide nucleation sites for mineral deposition, while fluoride contributes to the formation of more stable mineral phases [45]. The findings of the present study are consistent with previous studies reporting increased microhardness following the application of ReMin Pro to artificially demineralized enamel [33,45,46]. In a study by Melo et al. [47], clear signs of remineralization and mineral deposition were observed in the SEM analysis of ReMin Pro-treated samples, which is consistent with the SEM findings obtained in the present study.

An increase in enamel surface microhardness was also observed in the bioactive glass group, consistent with previous studies reporting that bioactive glass can promote mineral deposition on demineralized enamel surfaces [48,49]. Unlike ReMin Pro, the remineralization mechanism of bioactive glass involves ion release from the material followed by the formation of a mineral phase, representing a more gradual process [48]. In contrast, the hydroxyapatite and fluoride in ReMin Pro contribute to the mineralization process by promoting mineral deposition on the enamel surface [49].

When considered in light of these differences in the mechanisms of action, the 14-day experimental period and the use of a surface-sensitive assessment method such as surface microhardness testing may explain the differences in the magnitude of microhardness recovery observed among the materials. Consistent with this interpretation, SEM analysis showed a more heterogeneous distribution of surface improvement in the bioactive glass group. Previous studies have also reported that bioactive glass can increase mineral deposition on enamel surfaces, although its effects may vary depending on experimental conditions such as material concentration, application duration, and the assessment method employed [49].

Grape seed extract has attracted attention as a natural agent because of its polyphenolic constituents and reported antioxidant and antibacterial properties [50]. In the present study, its application resulted in a significantly greater increase in enamel surface microhardness compared with the control group, indicating that the extract contributed to microhardness recovery. Previous studies have similarly reported improvements in the microhardness of enamel and dentin following grape seed extract treatment, with the response varying according to the concentration used [27]. In addition, mineral deposition and structural improvement in enamel surfaces have been reported following its application [51], which is consistent with the morphological changes observed in the SEM analysis of the present study. The remineralization response may be related to interactions between the polyphenolic constituents of the extract and the mineralized enamel surface [50,52]. Nevertheless, the direct remineralizing mechanisms of grape seed extract remain less clearly defined than those of established fluoride-based agents. The relatively distinct response observed in the present study may therefore reflect differences in the mode of action of the extract compared with agents that directly provide or facilitate the availability of mineral ions. Furthermore, from a clinical perspective, when evaluating the broader applicability of GSE as a remineralizing agent, the potential for transient tooth discoloration associated with its polyphenolic content must also be considered [53].

This study has several limitations. First, the in vitro design cannot fully replicate the complex oral environment, including saliva, biofilm, dietary changes, masticatory forces, and host-related factors; therefore, the findings should be interpreted with caution when extrapolating to clinical conditions. Second, remineralization was evaluated using surface microhardness testing and scanning electron microscopy, which primarily provide information on surface changes. Although these methods allow for an assessment of changes in surface morphology and hardness, they do not directly provide information on subsurface mineral distribution, lesion depth, or the elemental composition of newly formed mineral deposits, as energy-dispersive X-ray spectroscopy (EDX) analysis was not performed. Third, the 14-day pH cycling model represents a simplified simulation of the oral environment and does not reflect long-term clinical behavior. In addition, scanning electron microscopy analysis was performed on one representative specimen per group, and the quantitative assessment was based primarily on surface microhardness. The present findings should be interpreted in light of these methodological considerations.

Future studies should investigate longer remineralization periods, different application protocols, and complementary techniques such as transverse microradiography, micro-computed tomography, X-ray diffraction (XRD), and energy-dispersive X-ray spectroscopy to provide a more comprehensive assessment of mineral changes. Although cost-effectiveness was not evaluated in the present study, factors such as material cost, application frequency, and treatment setting may influence the economic value of remineralizing agents. For example, previous research has shown that the cost-effectiveness of SDF may vary depending on caries activity and treatment setting [54]. Therefore, future studies should include detailed economic evaluations to better determine the clinical efficacy and cost-effectiveness of these agents. In addition, antibacterial activity may also contribute to controlling initial caries lesions [55]; thus, it should be evaluated alongside remineralization in future studies.

## 5. Conclusions

Within the limitations of this in vitro study, all tested remineralization agents significantly increased enamel surface microhardness compared with the control group. The greatest numerical increases were observed in the silver diamine fluoride, ReMin Pro, and bioactive glass groups, while the use of silver diamine fluoride and ReMin Pro resulted in significantly greater microhardness recovery than the use of grape seed extract. These findings support the potential of the tested agents to promote the remineralization of artificially demineralized enamel.

## Figures and Tables

**Table 1:** Composition of remineralization agents and manufacturers.

Material	Composition	Manufacturer
**Silver diamine fluoride (Riva Star)**	38% silver diamine fluoride, ammonia, potassium iodide, distilled water	SDI Limited, Bayswater, VIC, Australia
**ReMin Pro**	Nano-hydroxyapatite, xylitol, sodium fluoride, water, glycol, sodium carboxymethyl cellulose (CMC-Na), silicon dioxide	VOCO GmbH, Cuxhaven, Germany
**Bioactive glass (Bone-G Active-45S5)**	45S5 bioactive glass, water, glycol, sodium carboxymethyl cellulose (CMC-Na), silicon dioxide	Meta Bioengineering and R&D Services Inc., İstanbul, Türkiye
**Grape seed extract (Immu-Nat)**	Proanthocyanidins (OPCs), phenolic acids (caffeic acid, gallic acid), vitamin E (tocopherol), fatty acids (linoleic acid, oleic acid, palmitic acid), minerals (potassium, calcium, magnesium)	Immunat Herbal Pharmaceuticals Inc., Muğla Türkiye

**Table 2:** Comparison of surface microhardness values at baseline, after demineralization, and after remineralization.

	Baseline Measurement	Demineralization Measurement	Remineralization Measurement
	Mean ± SD	Mean ± SD	Mean ± SD
SDF	359.47 ± 31.14 ^ab^	238.18 ± 28.03	356.33 ± 27.43 ^a^
REMIN-PRO	366.31 ± 30.05 ^ab^	216.85 ± 27.54	339.99 ± 28.53 ^a^
BIOACTIVE GLASS	354.18 ± 41.93 ^ab^	221.71 ± 31.33	327.52 ± 41.39 ^a^
GSE	325.10 ± 37.09 ^a^	203.69 ± 27.35	286.59 ± 27.59 ^b^
CONTROL	369.48 ± 25.42 ^b^	213.78 ± 31.26	221.72 ± 23.58 ^c^
F	2.769	1.886	31.631
*p*	0.039 *	0.129	0.000 *

Values are presented as mean ± SD. Data were analyzed using one-way ANOVA followed by Bonferroni post hoc test. * Indicates a statistically significant difference among the initial, de-min-eralization, and remineralization measurements within each group (*p* < 0.05). Different superscript letters indicate statistically significant differences among groups (*p* < 0.05).

**Table 3:** Within-group pairwise comparisons of enamel microhardness measurements at baseline, after demineralization, and after remineralization.

Group	Baseline vs. Demineralization	Baseline vs. Remineralization	Remineralization vs. Demineralization
SDF	<0.001 *	0.968	<0.001 *
REMIN-PRO	<0.001 *	0.120	<0.001 *
BIOACTIVE GLASS	<0.001 *	0.285	<0.001 *
GSE	<0.001 *	0.026 *	<0.001 *
CONTROL	<0.001 *	<0.001 *	0.789

Pairwise comparisons within each group were performed using the Bonferroni post hoc test; * indicates a statistically significant difference *p* < 0.05.

## Data Availability

The data presented in this study are available from the corresponding author upon reasonable request.

## Abbreviations

ANOVA	Analysis of Variance
VHN	Vickers Hardness Number
CMC-Na	Sodium Carboxymethyl Cellulose
CPP-ACP	Casein Phosphopeptide–Amorphous Calcium Phosphate
EDS	Energy-Dispersive X-ray Spectroscopy
FEG	Field Emission Gun
IBM	International Business Machines
OPCs	Oligomeric Proanthocyanidins
SDF	Silver Diamine Fluoride
GSE	Grape Seed Extract
SEM	Scanning Electron Microscopy
SPSS	Statistical Package for Social Sciences
